# Effect of Whole or Ground Flaxseed Supplementation on Fatty Acid Profile, Fermentation, and Bacterial Composition in Rumen of Dairy Cows

**DOI:** 10.3389/fmicb.2021.760528

**Published:** 2021-11-12

**Authors:** Guoxin Huang, Liya Guo, Xiaofeng Chang, Kaizhen Liu, Wenhao Tang, Nan Zheng, Shengguo Zhao, Yangdong Zhang, Jiaqi Wang

**Affiliations:** ^1^College of Animal Sciences and Technology, Northeast Agricultural University, Harbin, China; ^2^Key Laboratory of Quality & Safety Control for Milk and Dairy Products of Ministry of Agriculture and Rural Affairs, Institute of Animal Sciences, Chinese Academy of Agricultural Sciences, Beijing, China; ^3^State Key Laboratory of Animal Nutrition, Institute of Animal Sciences, Chinese Academy of Agricultural Sciences, Beijing, China; ^4^Henan Institute of Science and Technology, Xinxiang, China

**Keywords:** flaxseed, α-linolenic acid, rumen fatty acid, rumen metabolism, bacteria

## Abstract

Flaxseed is rich in α-linolenic acid (ALA) and can increase omega-3 polyunsaturated fatty acid in the milk of dairy cows. However, the response of rumen fermentation to different forms of flaxseed supplementation is unknown. This study aimed to investigate the effect of different forms of flaxseed on the fatty acid profile, fermentation, and composition of bacteria in the rumen of dairy cows. In total, 30 Holstein dairy cows were selected and randomly assigned into three groups (10/group). Cows were fed a basal diet (control check; CK) or basal diets supplemented with either 1,500 g per day whole flaxseed (WF) or 1,500 g per day ground flaxseed (GF). The WF group had the highest ALA content in rumen fluid, whereas no difference was found between the CK and GF groups. However, the molar proportion of acetate increased in the WF and GF groups and was the highest in the GF group, and a similar trend was shown by propionate, isobutyrate, butyrate, isovalerate, and valerate (CK < WF < GF). The abundance of *Ruminococcaceae_*NK4A214*_group*, *Christensenellaceae_*R-7*_group*, and *Eubacterium_coprostanoligenes_group* also showed the same trend (CK < WF < GF). Different forms of flaxseed release ALA by different mechanisms in the rumen, and the molar proportions of volatile fatty acids and the bacterial composition were potentially influenced mainly by the amount of ALA released into the rumen.

## Introduction

As a source of omega-3 polyunsaturated fatty acid (n-3 PUFA), flaxseed is widely used to enhance levels of n-3 PUFA in milk production (Meignan et al., 2017; Brzozowska et al., 2018; Marino et al., 2019), particularly, α-linolenic acid (ALA; c9,c12,c15-C18:3). Flaxseed is rich in ALA, 18% of the total seed, and 53 to 56% of total fatty acids (Mustafa et al., 2002; Petit, 2003). In feeding experiments, whole (WF; Castillo-Lopez et al., 2018) and ground flaxseed (GF; Isenberg et al., 2019) are two important forms of dietary supplementation. However, previous studies reported that GF supplementation had a higher efficiency in increasing n-3 PUFA (mainly ALA) in milk (Silva et al., 2007; Petit and Cortes, 2010; Mesgaran et al., 2012). The n-3 PUFA (mainly ALA) in WF cannot be released as completely as GF because of the flaxseed shell. Most ALA in WF is excreted directly from the animal (Oba et al., 2009). To release ALA from WF, cows need to crush the flaxseed by tooth. Thus, in the rumen of ruminants, ALA in WF takes longer to be released. However, ALA in GF could be released into the rumen in a shorter time. Compared to a short and rapid release, a long release may lead to the continuous biological hydrogenation of ALA in the rumen. Therefore, the efficiency of transporting the ALA into milk is lower.

In the rumen, dietary PUFA can influence the composition of the microbiota (Fievez et al., 2007). Previous articles have reported that ALA was toxic to the growth of *Clostridium proteoclasticum*, *Butyrivibrio hungatei*, and *Eubacterium ruminantium* in the rumen (Maia et al., 2007). The release of ALA from either WF or GF is different and may influence the ruminal microbiota diversity. However, no studies have revealed the influence of ALA from different flaxseed on the microflora ruminal.

This study aimed to investigate the effect of WF or GF supplementation on rumen formation and bacterial community. We hypothesized that dietary supplementation with WF and GF may have a different effect on the rumen fatty acid profile and that the rumen fermentation pattern and the structure of bacteria may be influenced by the ALA released into the rumen.

## Materials and Methods

### Experimental Design and Treatments

This experiment was performed at Tianjin Fuyou Agricultural Technology Co., Ltd. (Tianjin, China). The animal protocol (protocol no. IAS 2019-28; date of approval: April 8, 2019) was approved by the Animal Care and Use Committee of the Institute of Animal Science, Chinese Academy of Agricultural Sciences, Beijing, China. Thirty primiparous Holstein dairy cows (90 ± 28 days in milk; 628 ± 103 kg body weight; 37.22 ± 2.60 kg of milk per day) were selected for the present study. Cows were randomly assigned into three groups and provided with a basal diet (control check; CK) or the basal diet supplemented with 1,500 g per day WF or 1,500 g per day GF, as shown in Table 1 (predicted feed intake: 22.3 kg per day and dry matter of flaxseed is 94.96%, thus flaxseed content in diet: 6.38%). The diets in this experiment were formulated and evaluated using the Feeding Standards of Dairy Cattle in China (Ministry of Agriculture of China (MOA). Feeding Standard of Dairy Cattle, NY/T 34-2004; MOA: Beijing, China, 2004). This experiment lasted for 5 weeks, and the cows were fed in a well-ventilated barn. The daily feeding times were 6:30 AM, 2:00 PM, and 9:30 PM using a total mixed ratio. In this experiment, the dry matter intake and milk yield showed no difference between the treatments.

**TABLE 1:** Composition of experimental diets (% DM basis).

Items	Treatments^a^		
	CK	WF	GF
Diet ingredient, % of DM			
Corn silage	21.51	21.86	21.86
Alfalfa hay	8.76	8.90	8.90
Alfalfa semidry silage	3.61	3.67	3.67
Wrapped straw	2.44	2.48	2.48
Corn	26.18	21.56	21.56
Corn flakes	2.77	2.82	2.82
Wool cotton seed	3.88	1.97	1.97
Orange peel granule	1.94	1.97	1.97
Soybean meal	12.42	11.8	11.8
Puffed soybeans	2.22	2.26	2.26
Cotton meal	3.12	3.17	3.17
DDGS	2.85	2.90	2.90
Bran	1.08	1.10	1.10
Fat powder	1.71	1.55	1.55
Flaxseed	0	6.38	6.38
Premix^b^	5.51	5.60	5.60
Total	100	100	100
Chemical, % of DM			
DM	51.18	51.27	53.58
NE_L_^c^, Mcal/kg of DM	1.91	1.94	1.94
CP	17.19	17.48	17.48
ADF	17.99	19.30	17.08
NDF	63.15	65.70	61.23
EE	4.59	8.52	8.50
FA, % of total FA reported			
C14:0	2.04	1.43	1.46
C16:0	76.05	67.02	68.04
c9-C16:1	0.03	0.03	0.04
C18:0	0.03	0.04	0.05
c9-C18:1	0.77	0.94	1.01
c9,c12-C18:2	18.86	9.84	9.49
c9,c12,c15-C18:3	2.10	20.60	19.80
C20:0	0.04	0.03	0.04
c11-C20:1	0.01	0.01	0.01
C22:0	0.04	0.03	0.04
C24:0	0.03	0.02	0.03

*DM, dry matter; NEL, net energy for lactation; CP, crude protein; ADF, acid detergent fiber; NDF, neutral detergent fiber; EE, ether extracts.*

*^a^CK, dairy cows fed a basal diet (without flaxseed); WF, dairy cows fed whole flaxseed diet (whole flaxseed 1,500 g per day); GF, dairy cows fed with ground flaxseed diet (ground flaxseed 1,500 g per day per cow).*

*^b^Premix (per kg of DM): a minimum of 313,500 IU of vitamin A, 104,500 IU of vitamin D, 5,000 IU of vitamin E, 780 mg of Cu, 780 mg of Fe, 780 mg of Mn, 3,900 mg of Zn, 30 mg of Se, 50 mg of I, and 65 mg of Co.*

*^c^Calculated value (based on China Standard NY/T 34; People’s Republic of China, 2004).*

### Sampling, Measurements, and Analysis

The rumen fluid was collected at 6:00 AM *via* esophageal tubing, which was described by Paz et al. (2016). Approximately, 100 mL of rumen fluid was collected and sieved through four cheesecloth layers. The first 10 mL was used to analyze the pH value using a pH meter (UB-10 pH meter, United States); the remaining rumen fluid was placed into eight 5-mL plastic cryotubes and stored in liquid nitrogen for further analysis. The rumen ammonia-N (NH_3_-N) concentration was determined according to the method by Feng and Gao (1993) and volatile fatty acid (VFA) concentration by using gas chromatography (Agilent Technologies, Santa Clara, CA, United States) (Hu et al., 2005). Ruminal fatty acid analysis was according to the method described by Dreiucker and Vetter (2011).

### DNA Extraction

The total rumen bacteria DNA was extracted by using cetyltrimethylammonium bromide (CTAB). Rumen fluid (1.5 mL) was centrifuged at 13,000 × *g* at 4°C for 10 min to collect the supernatant, which contains rumen bacteria. The supernatant and 800 μL CTAB (100 mM Tris-HCl, pH 8.0; 1.4 M NaCl; 20 mM EDTA; 2% CTAB) were mixed together and crushed by using a Mixer Mill MM 400 (Retsch, Haan, Germany) at a vibrational frequency of 30 m/s for 1 min. Next, the mixtures were incubated at 70°C for 20 min, and the supernatant was separated by centrifuge (13,000 × *g* for 10 min). Then, 700 μL of supernatant was mixed with 600 mL of phenolchloroform-isoamyl alcohol (volume 25:24:1), and 500 μL of the upper liquid was transferred into a new tube and mixed with 0.8 times the volume of isopropanol and centrifuged at 13,000 × *g* for 10 min at 4°C. The supernatant was removed from the tube and placed into 800 μL ethanol (70%) to precipitate the DNA. The quality of extracted DNA was evaluated through agarose gel electrophoresis and Nanodrop spectrometer (Thermo Scientific, Chicago, IL, United States). The average concentration of DNA was 396 ng/μL and the average of the 260/280 ratio was 1.88 for all samples.

### High-Throughput Sequencing of the 16S rDNA Gene

The V3 and V4 regions of 16S rDNA were selected for amplification by polymerase chain reaction (PCR) with the universal primers 341F (5′-CCTACGGGNGGCWGCAG) and 806R (5′-GGACTACHVGGGTATCTAAT) (Wang C. et al., 2019). The PCR incubation conditions were performed with KOD Polymerase (Toyobo, Osaka, Japan) at 95°C for 4 min, followed by 15 cycles at 95°C for 60 s, 60°C for 40 s, and 72°C for 60 s and a final extension at 72°C for 10 min. The PCR products were collected using an AxyPrep DNA Gel Extraction Kit (Axygen Biosciences, Union City, CA, United States).

### Sequencing Data Processing

The 16S rDNA amplicon sequencing was sequenced on an Illumina Novaseq 6000 platform (Guangdong Magigene Biotechnology, Guangzhou, China). QIIME (Quantitative Insights Into Microbial Ecology, version 1.9.1) was used in this experiment to analyze the sequences. According to the overlap relationship between pair-end reads, the sequence data were merged into one sequence tag. After that, UCLUST was used to cluster tags according to 97% similarity level. Naive Bayesian assignment algorithm of RDP Classifier (version 2.2) was used to identify those representative sequences against the Greengene database (version gg_13_5). α-Diversity (Ace, Chao, Good coverage, Shannon, Simpson, Sobs) and β-diversity PCoA (principal coordinate analysis) and ANOSIM were performed using QIIME (version 1.9.1). The sequence data have been submitted to the Sequence Read Archive of the NCBI under project PRJNA756791.

### Statistical Analysis

Homogeneity of variance and normal distribution for the variables of pH, NH_3_-N, VFAs, and fatty acid composition of the rumen were tested by the HOVTEST and UNIVARIATE of the SAS (version 9.4, SAS Institute, Inc., Cary, NC, United States) software. Then the data were analyzed using one-way analysis of variance models in SAS. The following statistical model was used:


(1) $$Y_{\text{ij}}\,=\mu +T_{\text{i}}+\varepsilon_{\text{ij}},$$

where *Y*_ij_ represents the observed dependent variables, μ is the overall mean, *T*_i_ is the effect of treatment, and ε_ij_ is the residual error. The significance level was declared at *P* < 0.05.

## Results

### Rumen Fatty Acid Profiles

The concentration of fatty acid in rumen fluid is shown in Table 2. The proportion of C6:0 (caproic), C8:0 (caprylic), C10:0 (capric), C12:0 (lauric), and C14:0 (myristic) was no different between treatments. However, supplementation with GF increased the concentration of C16:0 (palmitic) in the rumen, compared with the WF and CK groups. The concentration of C18:1-trans (elaidic acid) in rumens decreased in WF compared with the CK and GF groups (*P* = 0.018). However, the levels of ALA (*P* = 0.039) and total n-3 PUFA (*P* = 0.036) were greater in the WF group relative to CK and GF groups. Supplementation with WF tended to increase the concentration of EPA compared with the CK and GF groups, but there was no significant difference between them (*P* = 0.099).

**TABLE 2:** Effects of dietary whole or ground flaxseed supplementation on rumen fatty acid (g/100 g total fatty acid).

Items	Treatments^1^			
	CK	WF	GF	*P*-value
C6:0, caproic	0.61 ± 0.25	0.50 ± 0.23	0.51 ± 0.29	0.597
C8:0, caprylic	0.48 ± 0.31	0.50 ± 0.30	0.71 ± 0.33	0.230
C10:0, capric	3.80 ± 1.18	2.24 ± 1.62	2.08 ± 0.90	0.606
C12:0, lauric	2.16 ± 0.94	3.25 ± 1.04	3.12 ± 1.17	0.963
C14:0, myristic	11.07 ± 2.55	11.91 ± 2.16	9.41 ± 2.90	0.192
C16:0, palmitic	55.87 ± 5.09^b^	57.72 ± 2.74^b^	62.18 ± 5.05^a^	0.013
C18:0, stearic	6.69 ± 0.83	8.38 ± 2.53	7.16 ± 1.39	0.101
Other-C18:1^2^	0.18 ± 0.03	0.22 ± 0.05	0.25 ± 0.07	0.057
t9-C18:1, elaidic acid	0.04 ± 0.01^a^	0.03 ± 0.01^b^	0.04 ± 0.01^a^	0.018
c9,c12-C18:2, linoleic acid	1.07 ± 0.24	0.86 ± 0.32	1.22 ± 0.58	0.164
c9,c12,c15-C18:3, (ALA)	0.21 ± 0.06^b^	0.44 ± 0.26^a^	0.33 ± 0.19^ab^	0.039
c5,c8,c11,c14c17-C20:5 (EPA)	0.47 ± 0.52	0.85 ± 0.69	0.32 ± 0.38	0.099
Total n-3 PUFA	0.67 ± 0.50^b^	1.25 ± 0.67^a^	0.69 ± 0.33^b^	0.036
C22:0, docosanoic acid	0.03 ± 0.01	0.02 ± 0.01	0.02 ± 0.01	0.212
c3-C22:1, *cis*-3-Docosenoate	0.08 ± 0.02	0.06 ± 0.03	0.07 ± 0.04	0.334

*ALA, α-linolenic acid; EPA, eicosapentaenoic acid; n-3 PUFA, omega-3 polyunsaturated fatty acid.*

*^1^CK, dairy cows fed with control check diet (without flaxseed); WF, dairy cows fed with whole flaxseed diet (whole flaxseed 1,500 g per day); GF, dairy cows fed with ground flaxseed diet (ground flaxseed 1,500 g per day).*

*^2^Other-C18:1 = c8-C18:1, c6-C18:1, and c9-C18:1.*

*^a,b^Means in the same row with different superscripts differ significantly in terms of treatment effect.*

### Ruminal Fermentation Parameters

The indices of ruminal fermentation are shown in Table 3. Flaxseed supplementation had no influence on the rumen pH (*P* = 0.115), and no differences were found in ruminal NH_3_-N between the three groups (*P* = 0.630). The molar proportion of VFAs showed differences between the treatments. Compared to the CK group, the molar proportion of acetate increased in the WF and GF groups and was highest in the GF group (*P* < 0.001). In addition, propionate, isobutyrate, butyrate, isovalerate, and valerate showed the same trend CK < WF < GF (*P* < 0.05). Ground and WF supplementation could decrease the ratio of acetate to propionate (*P* < 0.001).

**TABLE 3:** Effects of dietary whole and ground flaxseed supplementation on rumen fermentation indexes.

Items	Treatments^1^			
	CK	WF	GF	*P*-value
pH	7.07 ± 0.14	6.94 ± 0.23	6.86 ± 0.26	0.115
NH_3_-N (mg/dL)	5.74 ± 0.96	6.53 ± 1.93	6.27 ± 2.39	0.630
Molar proportion, %				
Acetate	60.25 ± 1.58^a^	52.02 ± 1.85^b^	48.29 ± 2.61^c^	<0.001
Propionate	19.70 ± 1.7^b^	20.38 ± 1.32^ab^	21.88 ± 2.13^a^	0.028
Isobutyrate	1.81 ± 0.23^c^	3.05 ± 0.30^b^	3.71 ± 0.26^c^	<0.001
Butyrate	13.82 ± 1.33^c^	17.30 ± 1.13^b^	20.53 ± 1.04^a^	<0.001
Isovalerate	2.88 ± 0.30^c^	3.64 ± 0.42^b^	4.73 ± 0.44^a^	<0.001
Valerate	1.54 ± 0.17^c^	2.10 ± 0.14^b^	2.38 ± 0.11^a^	<0.001
Acetate:propionate ratio	3.08 ± 0.31^a^	2.39 ± 0.19^b^	2.40 ± 0.32^b^	<0.001

*NH_3_-N = ammonia-N.*

*^1^CK = dairy cows fed with control check diet (without flaxseed); WF = dairy cows fed with whole flaxseed diet (whole flaxseed 1,500 g per day); GF = dairy cows fed with ground flaxseed diet (ground flaxseed 1,500 g per day).*

*^a–c^Means in the same row with different superscripts differ significantly in terms of treatment effect.*

### Sequencing and α-Diversity Measures

A total of 3,134,077 high-quality sequences were generated in the partial 16S rRNA gene amplicon sequencing and an average of 103,622 ± 7,687 in the rumen. α-Diversity measures are shown in Table 4. All the indexes of α-diversity measures were not significantly different between the CK and WF groups (*P* < 0.05). However, compared with the CK and WF group, the Shannon (*P* < 0.001), Simpson (*P* = 0.004), and Ace (*P* = 0.039) indexes were lower in the GF group. The goods coverage value was highest in the GF group (*P* = 0.002) and was higher than 0.99 in all groups.

**TABLE 4:** Effects of dietary whole and ground flaxseed supplementation on α-diversity indexes of rumen bacteria.

Items	Treatments^1^			*P*-value
	CK	WF	GF	
Ace	1,960.48 ± 188.81^ab^	2,006.18 ± 101.02^a^	1,820.80 ± 173.69^b^	0.039
Chao	1,918.23 ± 167.97	1,973.29 ± 90.48	1,833.69 ± 166.70	0.118
Goods_coverage	0.9939 ± 0.0017^b^	0.9933 ± 0.0015^b^	0.9958 ± 0.0013^a^	0.002
Shannon	8.59 ± 0.09^a^	8.42 ± 0.15^a^	7.90 ± 0.46^b^	<0.001
Simpson	0.9934 ± 0.0013^a^	0.9929 ± 0.0011^a^	0.9829 ± 0.0121^b^	0.004
Sobs	1,687.30 ± 165.16	1,690.70 ± 88.42	1,554.60 ± 190.48	0.098

*^1^CK, dairy cows with control check diet (without flaxseed); WF, dairy cows with whole flaxseed diet (whole flaxseed 1,500 g per day); GF, dairy cows with ground flaxseed diet (ground flaxseed 1,500 g per day).*

*^a,b^Means in the same row with different superscripts differ significantly in terms of treatment effect.*

### Bacterial Composition

**FIGURE 1:**
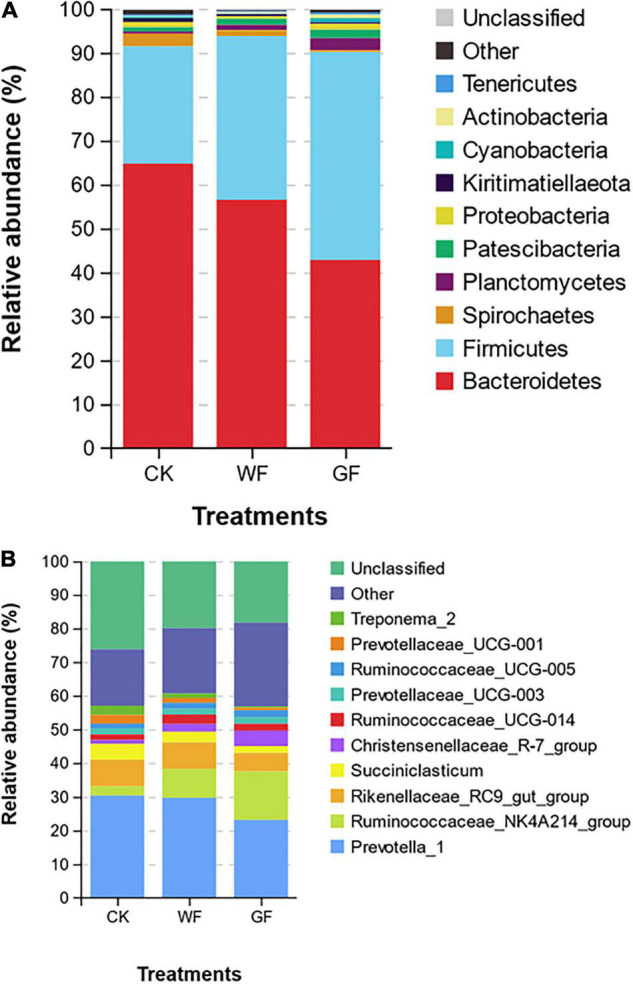
**(A)** Relative abundance of the bacterial community on the phylum level (only the top 10 abundant phyla are presented). **(B)** Relative abundance of bacterial communities on the genus level (only the top 10 abundant genera are presented). CK, dairy cows fed with control check diet (without flaxseed); WF, dairy cows fed with whole flaxseed diet (whole flaxseed 1,500 g per day); GF, dairy cows with ground flaxseed diet (ground flaxseed 1,500 g per day).

In the rumen, bacteria are the main microbe and are divided into many different phylum or genus. At the phylum level, 19 taxa were detected in the rumen. Figure 1A shows that the Bacteroidetes (CK: 64.77%, WF: 56.53%, GF: 42.84%), Firmicutes (CK: 26.69%; WF: 37.28%; GF: 47.31%), and Spirochaetes (CK: 2.90%; WF: 1.37%; GF: 0.49%) were the main phyla in all groups. Flaxseed supplementation reduced the abundance of Bacteroidetes (*P* < 0.001) and Spirochaetes (*P* < 0.001), whereas Firmicutes improved (*P* < 0.001) in rumen. At the genus level, a total of 354 genera were detected in the rumen. As shown in Figure 1B and Table 5, the unclassified sequences in CK, WF, and GF were 27.49%, 19.94%, and 19.29%, respectively, and *Prevotella_*1 (CK 30.41%; WF 29.76%; GF 23.19%) was the most abundant genera in rumen. The results of this experiment showed that feeding a GF diet could reduce the abundance of *Rikenellaceae_*RC9*_gut_group* (*P* = 0.042) compared with the CK and WF diet. The abundance of *Succiniclasticum* (*P* = 0.005), *Prevotellaceae_*UCG-001 (*P* < 0.001), *Treponema 2* (*P* < 0.001), and *Fibrobacter* (*P* < 0.001) showed a similar trend: CK > WF > GF. However, the abundance of *Ruminococcaceae_*NK4A214*_group* (*P* < 0.001), *Christensenellaceae_*R-7_*group* (*P* = 0.001), *Eubacterium coprostanoligenes* (*P* = 0.005), *Candidatus saccharimonas* (*P* = 0.002), the *p*-1088-a5 gut group (*P* < 0.001), and *Butyrivibrio*_2 (*P* = 0.047) showed a CK < WF < GF trend.

**TABLE 5:** Effects of dietary whole and ground flaxseed supplementation on rumen bacterial genera^1^.

Microbials	Treatments^2^			*P*-value
	CK	WF	GF	
*Prevotella_1*	30.41 ± 6.59	29.76 ± 7.06	23.19 ± 12.20	0.158
*Ruminococcaceae_NK4A214_group*	2.64 ± 1.56^c^	8.50 ± 3.39^b^	14.33 ± 9.07^a^	<0.001
*Rikenellaceae_RC9_gut_group*	7.92 ± 2.22^a^	7.87 ± 1.77^a^	5.47 ± 1.98^b^	0.015
*Succiniclasticum*	4.71 ± 2.21^a^	3.17 ± 1.18^b^	2.03 ± 1.44^b^	0.005
*Christensenellaceae_R-7_group*	1.15 ± 0.80^b^	2.31 ± 1.13^b^	4.61 ± 2.87^a^	0.001
*Prevotellaceae_UCG-001*	2.57 ± 0.48^a^	1.48 ± 0.65^b^	0.72 ± 0.36^c^	<0.001
*Treponema_2*	2.74 ± 0.96^a^	1.30 ± 0.66^b^	0.46 ± 0.30^c^	<0.001
*Candidatus_Saccharimonas*	0.73 ± 0.38^b^	1.20 ± 0.41^b^	1.91 ± 1.04^a^	0.002
*Eubacterium_coprostanoligenes_group*	0.85 ± 0.24^b^	1.42 ± 0.57^ab^	1.94 ± 0.99^a^	0.005
*Butyrivibrio_2*	0.64 ± 0.16^b^	1.33 ± 0.66^ab^	2.27 ± 2.33^a^	0.047
*Saccharofermentans*	0.73 ± 0.28^b^	1.11 ± 0.34^ab^	1.17 ± 0.65^a^	0.084
*p-1088-a5_gut_group*	0.28 ± 0.26^b^	0.54 ± 0.28^b^	1.63 ± 1.12^a^	<0.001
*Lachnospiraceae_NK3A20_group*	0.38 ± 0.10^b^	0.94 ± 0.40^a^	1.08 ± 0.85^a^	0.019
*Ruminococcus_2*	0.31 ± 0.17^b^	0.74 ± 0.50^ab^	0.95 ± 0.70^b^	0.027
*Schwartzia*	0.46 ± 0.55	1.00 ± 0.62	0.81 ± 1.32	0.414
*Lachnospiraceae_XPB1014_group*	0.19 ± 0.08^b^	0.51 ± 0.21^a^	0.70 ± 0.21^a^	<0.001
*Ruminococcus_gauvreauii_group*	0.21 ± 0.15^b^	0.44 ± 0.28^a^	0.52 ± 0.21^a^	0.012
*Ruminococcus_1*	0.59 ± 0.18^a^	0.28 ± 0.10^b^	0.28 ± 0.14^b^	<0.001
*Prevotellaceae_NK3B31_group*	0.42 ± 0.28^a^	0.34 ± 0.11^a^	0.16 ± 0.12^b^	0.013
*Anaerovorax*	0.40 ± 0.20^a^	0.26 ± 0.08^b^	0.20 ± 0.12^b^	0.015
*Fibrobacter*	0.81 ± 0.31^a^	0.07 ± 0.15^b^	0.03 ± 0.02^b^	<0.001
*Prevotellaceae_UCG-004*	0.34 ± 0.10^a^	0.20 ± 0.08^b^	0.12 ± 0.05^c^	<0.001
*Papillibacter*	0.36 ± 0.17^a^	0.09 ± 0.03^b^	0.09 ± 0.08^b^	<0.001
*Probable_genus_10*	0.13 ± 0.05	0.12 ± 0.06	0.08 ± 0.07	0.174
*Ruminococcaceae_V9D2013_group*	0.18 ± 0.18	0.11 ± 0.11	0.06 ± 0.07	0.122
*U29-B03*	0.14 ± 0.06^a^	0.09 ± 0.04^b^	0.05 ± 0.02^c^	<0.001
*Ruminiclostridium_6*	0.11 ± 0.05^a^	0.10 ± 0.07^ab^	0.06 ± 0.04^b^	0.100
*Moryella*	0.12 ± 0.04^a^	0.08 ± 0.02^b^	0.06 ± 0.04^b^	0.002
*Eubacterium_xylanophilum_group*	0.15 ± 0.09^a^	0.03 ± 0.04^b^	0.01 ± 0.02^b^	<0.001

*^1^Bacterial genus shown in this table met the following three terms: 1. Relative abundance greater than 0.10% (according to CK group); 2. Detected in at least 70% of animals in each group.*

*^2^CK, dairy cows fed with control check diet (without flaxseed); WF, dairy cows fed with whole flaxseed diet (whole flaxseed 1,500 g per day); GF, dairy cows with ground flaxseed diet (ground flaxseed 1,500 g per day).*

*^a–c^Means in the same row with different superscripts differ significantly in terms of treatment effect.*

### Principal Coordinate Analysis

**FIGURE 2:**
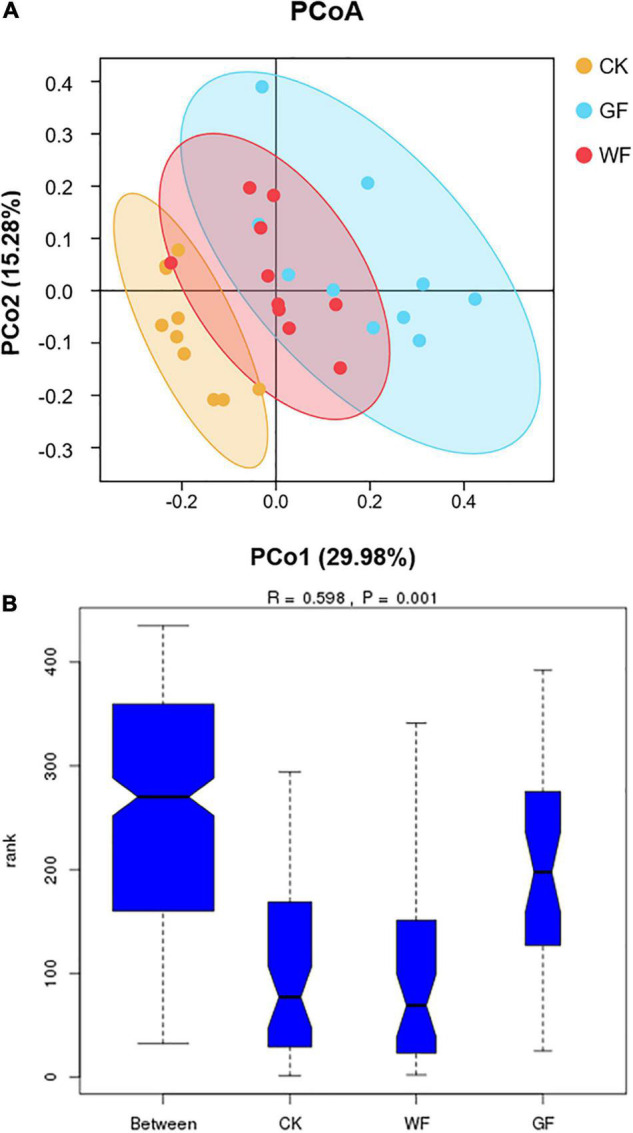
**(A)** Principal coordinate analysis of rumen bacterial samples; **(B)** similarity of bacterial genera among the three groups. CK, dairy cows fed with control check diet (without flaxseed); WF, dairy cows fed with whole flaxseed diet (whole flaxseed 1,500 g per day); GF, dairy cows fed with ground flaxseed diet (ground flaxseed 1,500 g per day).

We used PCoA to compare the OTU among the CK, WF, and GF groups (PCo1 = 29.98%; PCo2 = 15.28%). As shown in Figure 2A, a clear separation was observed in the plot of the CK and GF groups and could be well distinguished with the value of PCo1. On the PCo1 axis, most GF was distributed in the positive score values, whereas most CK was in the negative. However, it was difficult to distinguish between the CK and GF groups with the value of PCo2. At the same time, WF could not be separated from the CK and GF groups. Comparisons of ANOSIM test showed significant differences in genus level between CK, WF, and GF (*R* = 0.598; *P* = 0.001) (as shown in Figure 2B). This result revealed that a change in diet composition influenced the composition of the microorganisms in the rumen.

## Discussion

The ALA in WF and that in GF were released in different ways. The GF could release the ALA into the rumen directly, and approximately 4 h later, the PUFA concentration was back to normal (Baldin et al., 2018). In this experiment, rumen fluid was collected more than 4 h after feeding; thus, no difference was shown between the concentrations of ALA, EPA, and total n-3 PUFA in the CK and GF groups. However, because of the shell, the ALA in WF could only be released after rumination, and this release may last longer. Thus, higher concentrations of ALA, EPA, and total n-3 PUFA were found in the WF group, but not all WF can be broken or crushed through rumination, and therefore, many were excreted in the stools (Oba et al., 2009). Thus, higher concentrations of ALA, EPA, and n-3 PUFA in milk were found in the GF group, compared to the WF group.

In this study, flaxseed supplementation had no effect on rumen pH and NH_3_-N. Similarly, a previous study also reported that flaxseed oil supplementation had no effect on the pH and NH_3_-N in the rumen (Pi et al., 2019). However, supplementation with ALA can modify the molar proportion of VFAs in the rumen (Gao et al., 2016). VFAs are important metabolites that are produced by rumen fermentation and can provide 70% of the digestible energy required by the host (Bergman, 1990). Previous studies have reported that GF supplementation reduced the molar proportion of acetate and increased propionate in the rumen (Velez, 2012; Isenberg, 2014). The same result was also reported with WF supplementation in diet (Gutierrezgomez et al., 2020). However, a study by Kholif et al. (2018) found that GF supplementation in the diet enhanced the molar proportion of propionate but had no effect on acetate in the rumen. This might be attributed to the differences in animal species and flaxseed form. Kholif et al. (2018) chose goats and crushed flaxseed for their experiment, whereas this study used dairy cows and GF. The flaxseed form can influence the release of oil from seeds (Kholif et al., 2018). GF supplementation showed a greater influence on the molar proportion of VFAs in the rumen compared with WF supplementation. ALA in crushed flaxseed may release more slowly during digestion and fermentation and showed no effect on the molar proportion of acetate in the rumen. In this study, GF supplementation showed greater influence on the molar proportion of VFAs in the rumen compared to WF supplementation. Feeding flaxseed reduced the ratio of acetate to propionate, and this result was the same in a study by Castagnino et al. (2015). Flaxseed supplementation can shift rumen fermentation to propionate at the expense of acetate (Vargas et al., 2020). But no difference was shown in the ratio of acetate to propionate. In ruminants, rumen microbes play an essential role in shaping the VFAs in the rumen (Mcallister et al., 1994; Xue et al., 2019).

Diet is the main factor in shaping the bacterial communities in the rumen (Ley et al., 2008; Henderson et al., 2015). The addition of fatty acids, especially PUFA, could influence the microbial community (Enjalbert et al., 2017). GF supplementation could change the abundance of dominant phylum in the rumen compared to the CK and WF groups. Bacteroidetes species have a high correlation with enzymes involved in acetate and butyrate, and Firmicutes species are positive with ruminal butyrate (Deusch et al., 2017). Indeed, this experiment also observed that the lowest molar proportion of acetate and highest abundance of Bacteroidetes and the highest molar proportion of butyrate and the lowest abundance of Firmicutes were all found in the GF group. At the genus level, the relative abundances of bacteria *Treponema* 2, *Prevotellaceae_UCG*-001, and *Succiniclasticum* were significantly lower in the GF and WF groups than in the CK group, and lower in the GF group than in the WF group. The results also showed that *Treponema*_2 and *Prevotellaceae UCG*-001 contributed to higher acetate and propionate concentrations. Treponema species, a common bacterial group, can digest soluble fibers (Bekele et al., 2011); Treponema 2 belongs to this group. *Prevotellaceae_UCG*-001 belongs to the *Prevotellaceae* family, which has a positive association with digestive proteins and amino acids (Zhao et al., 2017; Zhu et al., 2019), and is unable to degrade fibers. However, *Prevotellaceae* has the ability to promote fiber degradation, when cocultured with cellulolytic bacteria (Morotomi et al., 2009; Rosenberg, 2014). Other studies have reported that *Succiniclasticum* was involved mainly in the fermentation of succinate to propionate (Gylswyk, 1995). In this study, our results also showed a correlation between *Succiniclasticum* and the molar proportion of propionate.

Previous research a high correlation between *Ruminococcaceae_*NK4A214*_group* and rumen biohydrogenation of 9c-C18:1 to C18:0 (Wang X. et al., 2019). *Ruminococcaceae_*NK4A214*_group*. *Ruminococcaceae_*NK4A214*_group* and *Eubacterium_ coprostanoligenes_group* all belong to *Ruminococcaceae*. In addition, *Ruminococcaceae* may play a predominant role in biohydrogenation in the rumen (Huws et al., 2011). In this study, the GF group had a higher abundance of Ruminococcaceae_NK4A214_group compared to the WF group. This can be associated with more ALA released in the rumen from GF supplementation. *Christensenellaceae_*R-7*_group* showed the same trend as *Ruminococcaceae_*NK4A214*_group* and *Eubacterium coprostanoligenes* between the treatments in this experiment. There are no studies that report a relationship between the *Christensenellaceae_*R-7*_group* and *Eubacterium coprostanoligenes* in rumen biohydrogenation, but many studies have found a positive correlation between the *Ruminococcaceae_*NK4A214*_group*, *Christensenellaceae_*R-7*_group*, and *Eubacterium coprostanoligenes* with milk yield (Tong et al., 2018; Sun et al., 2019). Although the three bacteria do not all belong to the same family (*Ruminococcaceae*: *Ruminococcaceae_*NK4A214*_group* and *Christensenellaceae_*R-7*_group*; *Christensenellaceae*: *Eubacterium coprostanoligenes*), they might belong to the same functional group (Morais and Mizrahi, 2019). The *Christensenellaceae_*R-7*_group* and *Eubacterium_coprostanoligenes_group* may promote rumen biohydrogenation alone or cooperate with others.

## Conclusion

Different forms of flaxseed released ALA differently in the rumen. WF released the ALA over a long time, whereas GF released ALA over a shorter time, which had a different effect on the composition of fatty acids in the rumen. The composition of microorganisms and the molar proportion of VFA in the rumen showed the same trend with different flaxseed form supplementation. Supplementation with GF changed the dominant bacterial at the phylum level and increased the abundance of the *Ruminococcaceae_*NK4A214*_group*, *Christensenellaceae_*R-7*_group*, and *Eubacterium coprostanoligenes groups*. In addition, the *Ruminococcaceae_*NK4A214_*group* contributed to rumen biohydrogenation, and the *Christensenellaceae*_R-7_*group* and *Eubacterium coprostanoligene*s groups may promote biohydrogenation. Further study using metagenomic and metatranscriptomic analyses of the microbial metabolic pathways or metabolites that may contribute to biohydrogenation is needed.

## Data Availability Statement

The datasets presented in this study can be found in online repositories. The names of the repository/repositories and accession number(s) can be found below: NCBI SRA; PRJNA756791.

## Ethics Statement

The animal study was reviewed and approved by Animal Care and Use Committee of the Institute of Animal Science, Chinese Academy of Agricultural Sciences. Written informed consent was obtained from the owners for the participation of their animals in this study.

## Author Contributions

GH: conceptualization, software, data curation, and writing – original draft. LG: methodology. XC: data curation. KL: software. WT: resources. SZ: formal analysis. YZ: writing – review and editing. NZ: supervision. JW: project administration. All authors contributed to the article and approved the submitted version.

## Conflict of Interest

The authors declare that the research was conducted in the absence of any commercial or financial relationships that could be construed as a potential conflict of interest.

## Publisher’s Note

All claims expressed in this article are solely those of the authors and do not necessarily represent those of their affiliated organizations, or those of the publisher, the editors and the reviewers. Any product that may be evaluated in this article, or claim that may be made by its manufacturer, is not guaranteed or endorsed by the publisher.
